# Conservative medical intervention as a complement to CDT for BCRL therapy: a systematic review and meta-analysis of randomized controlled trials

**DOI:** 10.3389/fonc.2024.1361128

**Published:** 2024-04-26

**Authors:** Chuyu Deng, Zhiguo Wu, Zijie Cai, Xiaoyan Zheng, Chunzhi Tang

**Affiliations:** ^1^ Clinical Medical College of Acupuncture, Moxibustion and Rehabilitation, Guangzhou University of Chinese Medicine, Guangzhou, China; ^2^ School of Rehabilitation Sciences, Southern Medical University, Guangzhou, China

**Keywords:** breast cancer-related lymphedema, BCRL, meta-analysis, systematic review, adjunctive therapy

## Abstract

**Background:**

The effect of first-line complex decongestive therapy (CDT) for breast cancer-related lymphedema (BCRL) depending on various factors forces patients to seek additional treatment. Therefore, this meta-analysis was conducted to evaluate the effect of different conservative medical interventions as a complement to CDT. This is the first meta-analysis that includes various kinds of conservative treatments as adjunctive therapy to get broader knowledge and improve practical application value, which can provide recommendations to further improve BCRL patients’ health status.

**Methods:**

RCTs published before 18 December 2023 from PubMed, Embase, Cochrane Library, and Web of Science databases were searched. RCTs that compared the effects of conservative medical intervention were included. A random-effects or fixed-effects model was used based on the heterogeneity findings. Study quality was evaluated using the Cochrane risk of bias tool.

**Results:**

Sixteen RCTs with 690 participants were included, comparing laser therapy, intermittent pneumatic compression (IPC), extracorporeal shock wave therapy (ESWT), electrotherapy, ultrasound, diet or diet in combination with synbiotic supplement, traditional Chinese medicine (TCM), continuous passive motion (CPM), and negative pressure massage treatment (NMPT). The results revealed that conservative medical intervention as complement to CDT had benefits in improving lymphedema in volume/circumference of the upper extremity [SMD = −0.30, 95% CI = (−0.45, −0.15), *P* < 0.05, *I* ^2 =^ 51%], visual analog score (VAS) for pain [SMD = −3.35, 95% CI (−5.37, −1.33), *P* < 0.05, *I* ^2 =^ 96%], quality of life [SMD = 0.44, 95% CI (0.19, 0.69), *P* < 0.05, *I* ^2 =^ 0], and DASH/QuickDASH [SMD = −0.42, 95% CI (−0.70, −0.14), *P* < 0.05, *I* ^2 =^ 10%] compared with the control group. Subgroup analysis revealed that laser therapy and electrotherapy are especially effective (*P* < 0.05).

**Conclusion:**

Combining conservative medical interventions with CDT appears to have a positive effect on certain BCRL symptoms, especially laser therapy and electrotherapy. It showed a better effect on patients under 60 years old, and laser therapy of low to moderate intensity (5–24 mW, 1.5–2 J/cm^2^) and of moderate- to long-term duration (≥36–72 sessions) showed better effects.

**Systematic review registration:**

https://www.crd.york.ac.uk/PROSPERO/display_record.php?RecordID=354824, identifier CRD42022354824.

## 1 Background

Breast cancer (BC) is the most frequently diagnosed cancer among women around the world although the incidence rate continues to rise and the overall mortality rate has declined with the advancement of early screening and diagnosis in many high-income countries (1). As the 5-year survival rate is almost 90% (2), there is an increasing number of BC survivors suffering long-term complications brought by surgery and radiation-related therapeutic exposure. BC-related lymphedema (BCRL) is one of the most seen complications among the survivors, with up to 40% of BC survivors suffering from upper extremity complications especially if lymphedema axillary lymph node procedures are applied (3). BCRL is associated with swelling in the limbs, persistent inflammation, pain, numbness, and restricted mobility (4), causing great distress in physical and mental function, and patients consider it as a medical burden (5, 6).

Since BCRL remains both incurable and debilitating (7), it is still challenging to confirm effective and safe therapy for patients. Currently, the first-line therapy for lymphedema is complex physical therapy/complex decongestive therapy (CDT), comprised of two phases: the first phase includes manual lymphatic drainage (MLD), compression therapy (bandages/garments/pumps), remedial exercise, and skin and nail care (8); the second phase includes lifelong self-care maintenance. Even though CDT has been proven as the most widely used treatment for lymphedema, its effectiveness depends greatly upon the therapist’s experience and overall patient compliance with the complex self-care requirement (9), which may cause low adherence in the long term because of repeated and tedious procedures. Furthermore, using compression therapy alone shows limited benefit to edema over a long-term period (10). The benefits of CDT will be greater if applied in the early lymphedema stage (stage I) (11) or less than 1 year of lymphedema duration (12). The severe condition of BCRL might hinder the effect of CDT, thus forcing patients to seek additional treatment (13), e.g., surgical treatment such as lymphaticovenular anastomosis (10, 14) and various conservative treatments to better improve the overall status of BCRL patients. Due to these limitations, a multidisciplinary approach and additional treatment strategies to treat BCRL systematically are necessary to be explored to optimize treatment efficiency and contribute to a more complete and efficient treatment, improving the quality of life as well as the adherence of the survivors (15).

To date, no studies have investigated the effectiveness of combinations of various conservative medical interventions and CTD. Our study was therefore designed to evaluate the effect of conservative interventions combining CDT in improving the symptoms of BCRL patients, and the results may help practitioners choose more efficient treatments. The hypothesis is that a combination of certain conservative interventions in these patients would improve the symptoms more significantly compared with CDT alone.

## 2 Methods

### 2.1 Eligibility criteria

All available randomized controlled trials (RCTs) met the following criteria: 1) types of studies: RCTs; 2) types of participants: women patients with BCRL and no restrictions on their BC and lymphedema stage, nationality, and age; 3) types of intervention: conservative treatment including physical therapy/oral supplements. Standard care (ST) was defined as any combination of skin care, exercises, compression method, and part of CDT. Giving the patients with only health education or guidance will be defined as none; 4) types of outcomes: primary outcomes were changes in edema, such as volume/circumference of the arm (lower scores mean better effect). Secondary outcomes included quality of life (QoL); VAS for pain; disabilities of the arm, shoulder, and hand (DASH/QuickDASH); and grip strength; and 5) language: English.

The exclusion criteria were as follows: 1) case reports, reviews, study protocols, conference abstracts, commentaries, and letters; 2) duplicate articles; 3) animal experiments; 4) studies that used surgical intervention and oral medicine (such as diosmin) and compared instruments like bandages/kinesio taping/garments aiming to compare different brands; studies that compared exercises were also excluded because our focus was on medical practice rather than self-care practice and studies that used intervention in the control group other than CDT/standard care/none; 5) any study design except RCT; 6) unavailable original full text; 7) language except English; and 8) studies with outcome only shown in graphs without concrete data form and failure to contact the authors to obtain the data.

### 2.2 Information sources and search strategy

Four relevant English literature databases (Embase, Cochrane Library, Web of Science, PubMed) were searched for all relevant citations published until 18 December 2023. We established search strategies that combined Medical Subject Headings (MESH term) and random words related to BCRL, interventions of interest (treatment/therapy), and RCTs. Furthermore, the reference lists of the included studies were manually reviewed to look for additional relevant manuscripts. The specific search strategies are shown in Appendix 1.

### 2.3 Selection process

Two reviewers (CYD, ZGW) independently screened the titles and abstracts of identified citations and full texts of potentially eligible studies. Disagreements were resolved by discussion or third-party (XYZ) adjudication when necessary.

### 2.4 Data collection and data items

Two reviewers (CYD, ZJC) independently extracted the study data, including the name of the first author, publication year, participant characteristics (country and age), sample size, intervention and the comparison treatments, baseline, course of treatment, outcomes, adverse events (AEs), and dropout. Lymphedema volume was measured as volume calculated from the circumference, water displacement and bioimpedance spectroscopy. Two researchers (CYD, ZJC) checked the extracted data for consistency, and a third researcher (XYZ, CZT) arbitrated any dispute.

### 2.5 Study risk of bias assessment

The Cochrane Risk of Bias Assessment tool (Cochrane Collaboration) was used to assess the risk of bias. The following types of bias were assessed: 1) random sequence generation, 2) allocation concealment, 3) blinding of participants and personnel, 4) blinding of outcome assessment, 5) incomplete outcome data, 6) selective reporting, and 7) other bias. Each item was classified into three types: low risk, high risk, and unclear risk. The quality of the included trials was evaluated by two reviewers (CYD, ZJC) independently, and disagreements were resolved by a third researcher (ZGW).

### 2.6 Effect measures

Continuous data were expressed as the mean ± standard deviation (SD) and summarized as a mean difference (MD) or standardized mean difference (SMD). Considering the primary outcomes reflected by the volume (ml/cm^3^) or circumference (cm) of the arm, the different units can cause great differences in data size; therefore, SMD with 95% confidence intervals (CIs) was used to analyze the primary outcome.

Heterogeneity among the studies was detected using *P* and *I* ^2^ statistics. A random-effects model was adopted when heterogeneity was observed (*P* < 0.05 and/or *I* ^2^ > 50%); otherwise, a fixed-effects model was adopted. If the pooled result included clinical heterogeneity, subgroup analysis was performed to search for the source of heterogeneity. Clinical heterogeneity was defined as differences in participants, treatment, outcome characteristics, or research setting.

All data were analyzed using the Excel 2016 (Microsoft) and RevMan 5.3 (Cochrane Collaboration, Oxford, UK) software.

## 3 Results

### 3.1 Study selection

**Figure 1:**
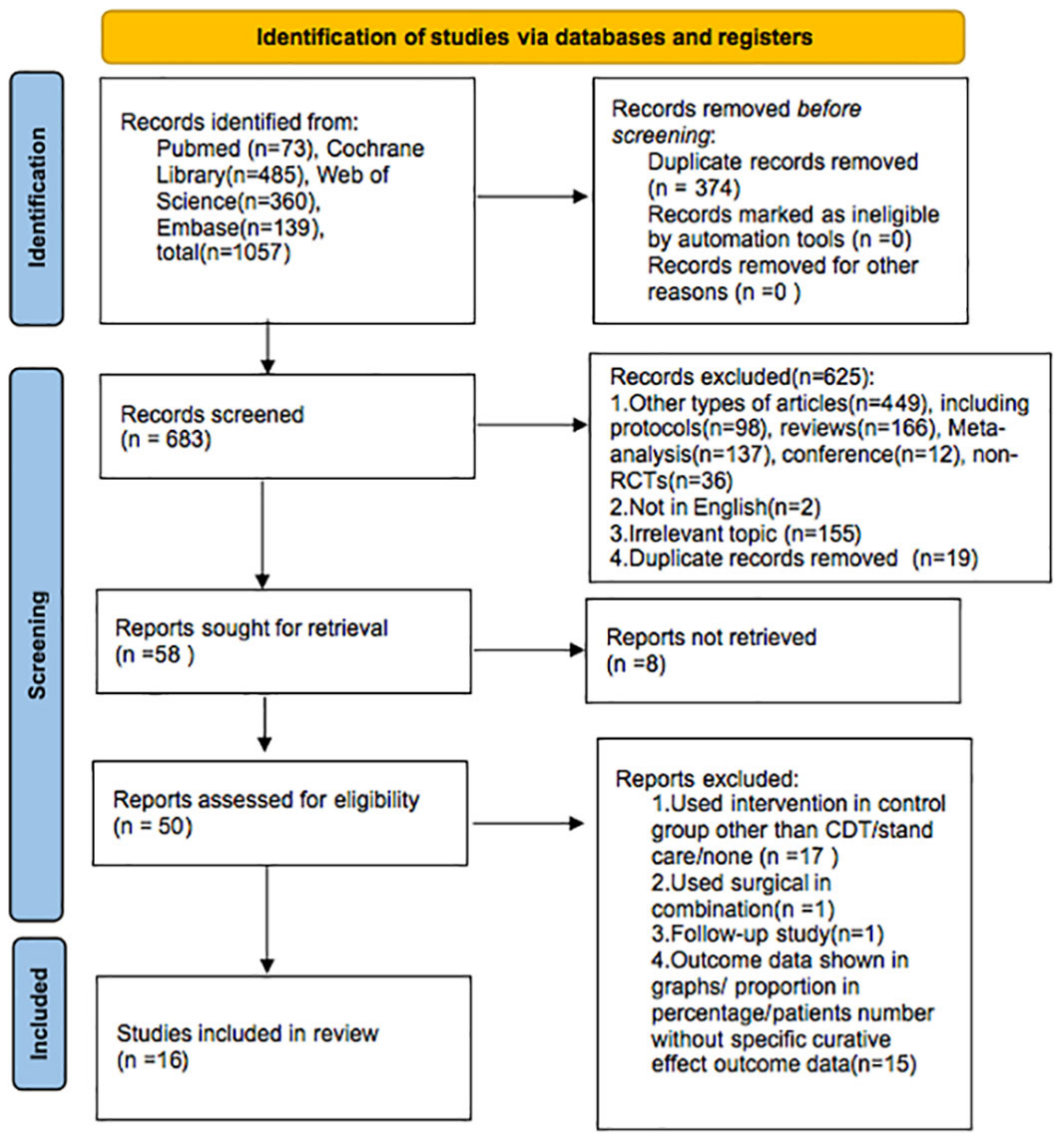
The PRISMA flow diagram.

The PRISMA flow diagram (**Figure 1**) displays the selection process. We found 1,057 articles by searching the databases (PubMed, Cochrane Library, Web of Science, Embase). After excluding 374 duplicate articles, two researchers conducted independent review and exclusion. Based on the title and abstract, 625 articles did not meet the selection criteria and were excluded, and 8 reports could not be retrieved.

Of the remaining 50 papers, 34 were excluded for the following reasons: in 17 studies, the control intervention was not CDT/standard care/none; 1 study used surgical intervention as combination; 1 study focused on follow-up research; and 15 articles did not report relevant concrete outcomes; specifically, the outcome data were shown in graphs/proportion in percentage/patients’ number without specific curative effect outcome data. Finally, 16 articles were included in our analysis.

### 3.2 Study characteristics

A total of 16 randomized controlled trials with 690 participants were ultimately included in this meta-analysis, of which 4 studies (16–19) adopted laser therapy; 3 studies (20–22) adopted IPC; 2 studies (23, 24) adopted ESWT; 1 study with three arms adopted electrotherapy (25) and ultrasound (25); 1 study with three arms adopted diet (26) or diet combined with a synbiotic supplement (26); 3 studies adopted the traditional Chinese medicine (TCM) method including sliding cupping (27), acupuncture (28), and Tuina in combination with moxibustion (29); 1 study adopted continuous passive motion (CPM) (30); and 1 study adopted negative pressure massage treatment (NMPT) (31).

Four RCTs (21, 22, 24, 30) were conducted in Turkey, three RCTs (20, 28, 31) were conducted in the USA, two RCTs (25, 26) were conducted in Iran, two RCTs (16, 19) were conducted in Egypt, three RCTs (17, 27, 29) were conducted in China, one RCT was conducted in Germany (18), and one RCT was conducted in Korea (23).

The results of the 16 RCTs involved changes in arm circumference (16, 23–25, 27–29) and/or volume (17–20, 22, 24–26, 29–31). The secondary outcomes in the included RCTs were VAS for pain (18, 21, 23, 25), quality of life (18, 26, 30), DASH (17, 30, 31) or QuickDASH (21, 23, 24), grip strength (16, 18, 21), bioimpedance (28, 31), BMI (26), range of motion (ROM) (16, 19, 20) of the shoulder, tissue resistance (17, 20), and skin thickness (23, 27). The summarized characteristics of the 16 RCTs are presented in **Table 1**.

**Table 1:** Characteristics of the included studies.

No.	References	Country	Age	Sample size (I/C)	Baseline	Outcomes	Course	AE	Dropout
1	Ahmed 2011 (16)	Egypt	I: 54.76 ± 3.33 C: 53.36 ± 3.56	LLLT (25)/ST (25)	2~8 cm interlimb circumference difference	1. Circumference 2. Grip strength 3. ROM	3 times/week for 12 weeks	UK	I: 4 withdrawn [cellulitis (*n* = 1), poor adherence (*n* = 3)] C: 4 withdrawn [cellulitis (*n* = 2), poor adherence (*n* = 2)]
2	Bao 2018 (28)	USA	I: 65 (54, 71) C: 58 (49, 70)	Acupuncture (40)/none (42)	>2 cm interlimb circumference difference in at least one of two sites (10 cm above or 5 cm below the olecranon process). Lymphedema diagnosed as stage II	1. Circumference 2. Bioimpedance	Twice/week for 6 weeks	Bruises 45 (58%) Hematoma 2 (2.6%) Pain 2 (2.6%) Skin infection 1 (1.3%)	I: *N* = 4 (lost to follow-up (*n* = 1), withdrew due to AE (*n* = 2), withdrew consent (*n* = 2)] C: Withdrew consent (*N* = 5)
3	Cebicci 2021 (24)	Turkey	I: 51.61 ± 6.6 C: 57.90 ± 6.9	ESWT (11)/CDT (12)	No description	1. Volume 2. Circumference 3. QuickDASH	3 times/week for 4 weeks	UK	I: *N* = 1 (1 lost to follow-up and withdrawn from the study) C: *N* = 2 (1 discontinued the treatment, 1 lost to follow-up)
4	Hemmati 2022 (25)	Iran	I: 48.96 ± 10.12 C: 49.13 ± 10.5	Electrotherapy + CDT (13)/CDT (13)	>2 cm interlimb circumference difference and/or >10% difference in volume between upper extremities	1. Volume 2. Circumference 3. VAS for pain	5 times/week for 2 weeks	UK	0
5	Hemmati 2022 (25)	Iran	I: 49.32 ± 10.15 C: 49.13 ± 10.5	Ultrasound + CDT (13)/CDT (13)	>2 cm interlimb circumference difference and/or >10% interlimb volume difference	1. Volume 2. Circumference 3. VAS for pain	5 times/week for 2 weeks	UK	0
6	Khalaf2012 (19)	Egypt	I: 49.13 ± 2.58 C: 48.66 ± 2.31	He–Ne laser + CDT (15)/CDT (15)	No description	1. Volume 2. ROM	3 times/week for 6 months	UK	0
7	Kizil 2018 (30) 50	Turkey	I: 55.50 (40–73) C: 58.00 (35–75)	CPM + CDT (16)/CDT (16)	>2 cm interlimb circumference difference	1. Volume 2. DASH 3. QoL (FACT-B4)	Once/day for 15 consecutive days	UK	I: *N* = 2 (2 lost to follow-up)
8	Kyeong 2020 (23)	Korea	I: 53.13 ± 10.85 C: 52.24 ± 8.60	ESWT + CDT (15)/CDT (15)	>2 cm interlimb circumference difference and a volume difference >200 ml	1. Volume 2. Circumference 3. VAS for pain 4. QuickDASH 5. Skin thickness	Twice/week for 3 weeks	No AEs occurred	0
9	Lampinen 2021 (31)	USA	I: 64.24 ± 13.69 C: 60.34 ± 10.65	NPMT (15)/MLD (13)	>150 ml interlimb volume difference, ≥2 cm interlimb circumference difference at any of the measured locations, or a lymph edema index (L-Dex) score of ≥7.1	1. Volume 2. L-Dex 3. DASH	2–3 times/week for 4–6 weeks, 12 sessions in total	No AEs occurred	I: *N* = 2 (1 fracture, 1 skin problem) C: *N* = 2 (1 leukemia, 1 commute challenge)
10	Lau 2009 (17)	China	I: 50.9 ± 8.6 C: 51.3 ± 8.9	LLLT (11)/none (10)	>200 ml interlimb volume difference	1. Volume 2. Tissue resistance 3. DASH	12 sessions of LLLT in 4 weeks	UK	0
11	Storz 2017 (18)	Germany	I: 61.06 ± 9.66 C: 59.37 ± 10.16	LLLT (20)/none (20)	In both groups, median pain intensity was 4 at baseline. QoL measured using MMSQ and MQoL was slightly higher in the active laser group than in the sham group (82.75 vs. 79.88 and 6.43 vs. 6.28). Regarding grip strength, both groups were nearly identical. Median limb volume difference was higher in the placebo group (160.46 ml/cm³) than in the active laser group (91.63 ml/cm³); however, this difference was not statistically significant.	1. Volume 2. QoL 3. Grip strength 4. VAS for pain	2 times/week for 4 weeks	No AEs occurred	I: *N* = 3 (2 withdrawn: poor adherence; 1 withdrew: not interested) C: *N* = 1 (withdrawn for poor adherence)
12	Szuba 2002 (20)	USA	I: 68.8 ± 9.11 C: 65 ± 10.8	CDT + IPC (12)/CDT (11)	>20% interlimb volume difference	1. Volume 2. Tissue resistance 3. ROM	Once/day for 10 days	No AEs occurred	0
13	Tastaban 2020 (21)	Turkey	I: 53.0 (43.0–58.0) C: 55.0 (48.0–58.0)	CDT + IPC (38)/CDT (38)	>2 cm interlimb circumference difference or >10% interlimb volume difference	1. Volume 2. QuickDASH 3. VAS for pain 4. Grip strength 5. Depression	5 days/week for 4 weeks	UK	0
14	Uzkeser 2015 (22)	Turkey	I: 55 (42–75) C: 56 (37–75)	CDT + IPC (16)/CDT (15)	>2 cm interlimb circumference difference or >10% interlimb volume difference	1. Volume	5 times/week for 3 weeks	UK	I: *N* = 1 (move to another city)
15	Vafa 2020 (26)	Iran	I: 53.80 ± 1.42 C: 53.24 ± 1.5	Diet + synbio + CDT (45)/CDT (45)	Stage I or II lymphedema	1. Volume 2. BMI 3. QoL	Once for 10 weeks	No AEs occurred	I: *N* = 4 [long distance from residence to the clinic (*n* = 1); follow-up with other physicians (*n* = 2); and unwillingness to continue the study (*n* = 1)] C: *N* = 4 (long distance from residence to the clinic (*n* = 2), unwillingness to continue the study (*n* = 2)]
16	Vafa 2020 (32)	Iran	I: 52.41 ± 1.19 C: 53.24 ± 1.5	Diet + CDT(45)/CDT (45)	Stage I or II lymphedema	1. Volume 2. BMI 3. QoL	Once/day for 10 weeks	No AEs occurred	I: *N* = 6 [recurrent disease (*n* = 1); long distance from residence to the clinic (*n* = 3); follow-up with other physicians (*n* = 1); and unwillingness to continue the study (*n* = 1)] C: *N* = 4 [long distance from residence to the clinic (*n* = 2), unwillingness to continue the study (*n* = 2)]
17	Xiong 2019 (27)	China	I: 53.43 ± 11.87 C: 52.47 ± 11.27	Sliding cupping (30)/CDT (30)	Sliding cupping: 15 mild degree of edema (difference <3 cm), 10 moderate edema (3~5 cm), and 5 cases of severe edema (≥5 cm) CDT: 16 patients showed mild edema, 10 patients were moderate, and 4 patients were severe	1. Circumference 2. Skin thickness	Once/day for 14 days	UK	0
18	Wang 2023 (29)	China	I: 58.45 ± 5.92 C: 59.30 ± 7.06	Tuina + Moxi/CDT	≥2 cm interlimb circumference difference or ≥120 ml interlimb volume difference	1. Volume 2. Circumference 3. VAS for swelling	Tuina for 20 min + moxibustion for 20 min, twice/week for 4 weeks	No AEs occurred	I: *N* = 3 [intolerance to moxibustion odor (*n* = 2), pneumonia (*n* = 1)] C: *N* = 2 (loss to follow-up)

No., number; I, intervention; C, control; BCRL, breast cancer-related lymphedema; LLLT, low-level laser therapy; Moxi, moxibustion; CDT, complex decongestive therapy; ESWT, extracorporeal shock wave therapy; IPC, intermittent pneumatic compression; CPM, continuous passive motion; NMPT, negative pressure massage treatment; AE, adverse event; UK, unknown.

### 3.3 Risk of bias in the studies

**Figure 2:**
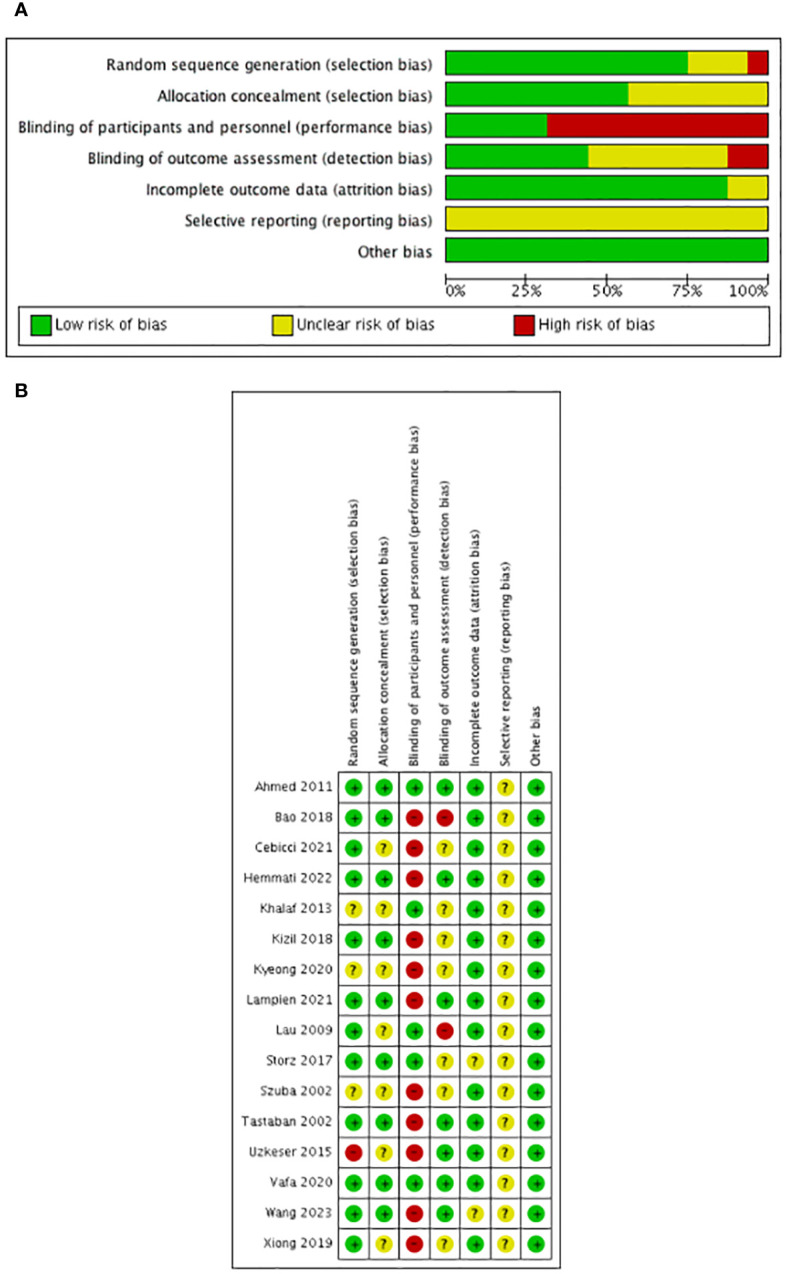
Risk of bias. **(A)** Overall quality assessment. **(B)** Individual quality assessment.

The risk of bias assessment is shown in **Figure 2**. All the included trials mentioned randomization: 12 studies described the randomization method in detail such as the random number table (27), block randomization (24, 26), block permutation method (25), random number table using block randomization (30), computer-generated program (16, 18, 21, 28, 29, 31), and the Bebbington method (17) and were defined as low risk of bias; 3 studies (19, 20, 23) had no concrete description of randomization and were defined as unclear risk of bias; and 1 study (22) randomized the patients according to admittance time and was defined as high risk of bias.

Furthermore, six studies (16, 18, 21, 28, 29, 31) used a computer-generated program in randomization to ensure the allocation concealment; two studies (25, 29) used sequentially numbered, sealed, and opaque envelopes; and one study (30) used an uninvolved researcher to assign patients. These studies were defined as low risk. The rest of the studies did not describe the allocation concealment and were defined as unclear risk.

As for performance blinding, blinding of the treating physiotherapist and patients in some interventions such as ESWT (24), IPC (20–22), CPM (30), NPMT (31), electrotherapy (25), ultrasound (25), acupuncture (28), sliding cupping (27), and Tuina in combination with moxibustion (29) was impossible considering the nature of the studies and according to the description of the authors; therefore, these were defined as high risk. Laser therapy (16–18, 31) (both active and placebo laser devices were similar in terms of weight, emitted sounds, and optical appearance to guarantee strictly controlled double-blinded conditions) and capsules (26) (the placebo capsule was comprised of lactose and was equal to the synbiotic capsule in terms of appearance, color, shape, size, smell, taste, and packaging) were performed in a blinded manner according to the description of the authors and were defined as low risk.

As for assessment blinding, six studies (16, 21, 22, 25, 29, 31) had a clear description of the blinding of outcome assessments and were defined as low risk. Two studies (17, 28) mentioned research staff not being blinded to the treatment group and were defined as high risk. Eight studies (18–20, 23, 24, 26, 27, 30) did not mention blinding in assessment and were defined as unclear risk.

Nine studies (16, 18, 22, 24, 26, 28–31) reported dropouts with clear reasons, and seven studies (17, 19–21, 23, 25, 27) reported no dropouts. We assessed the risk of attrition bias in all these studies as low risk.

As for selective reporting, although all the included studies reported all outcomes in the methods section, the protocol was not available and was defined as unclear risk.

Furthermore, we assessed the other risks as low considering that the whole design of most of the included studies was relatively formal.

### 3.4 Primary outcome: arm volume/circumference change

#### 3.4.1 Volume/circumference change and subgroup analysis

Of the 15 included studies, 4 studies (23–25, 29) reported both volume and circumference change of the limbs, 9 studies (17–22, 26, 30, 31) reported only volume change, and 3 studies (16, 27, 28) reported only circumference change. Considering the different units of volume (cm^3^) or circumference (cm) may result in great differences in data size; standardized mean difference (SMD) with 95% confidence intervals (CIs) was used.

**Figure 3:**
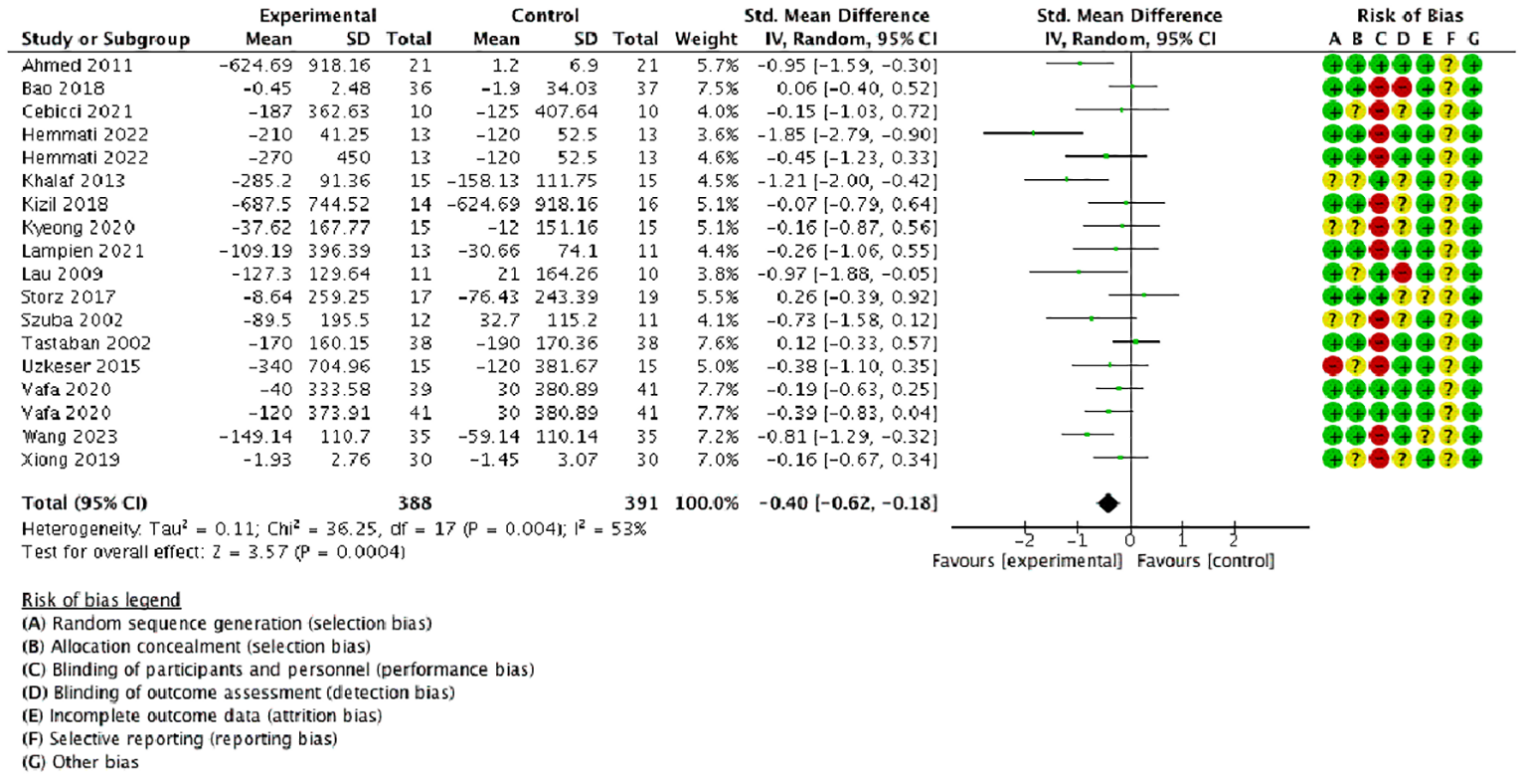
Forest plot of volume/circumference change.

The results revealed that conservative medical intervention as a complement to CDT had benefits in improving lymphedema in volume/circumference of the upper extremity compared with that of the control group [SMD = −0.40, 95% CI = (−0.62, −0.18), *P* < 0.05, *I* ^2 =^ 53%] (**Figure 3**).

#### 3.4.2 Subgroup analysis

##### 3.4.2.1 Age

**Figure 4:**
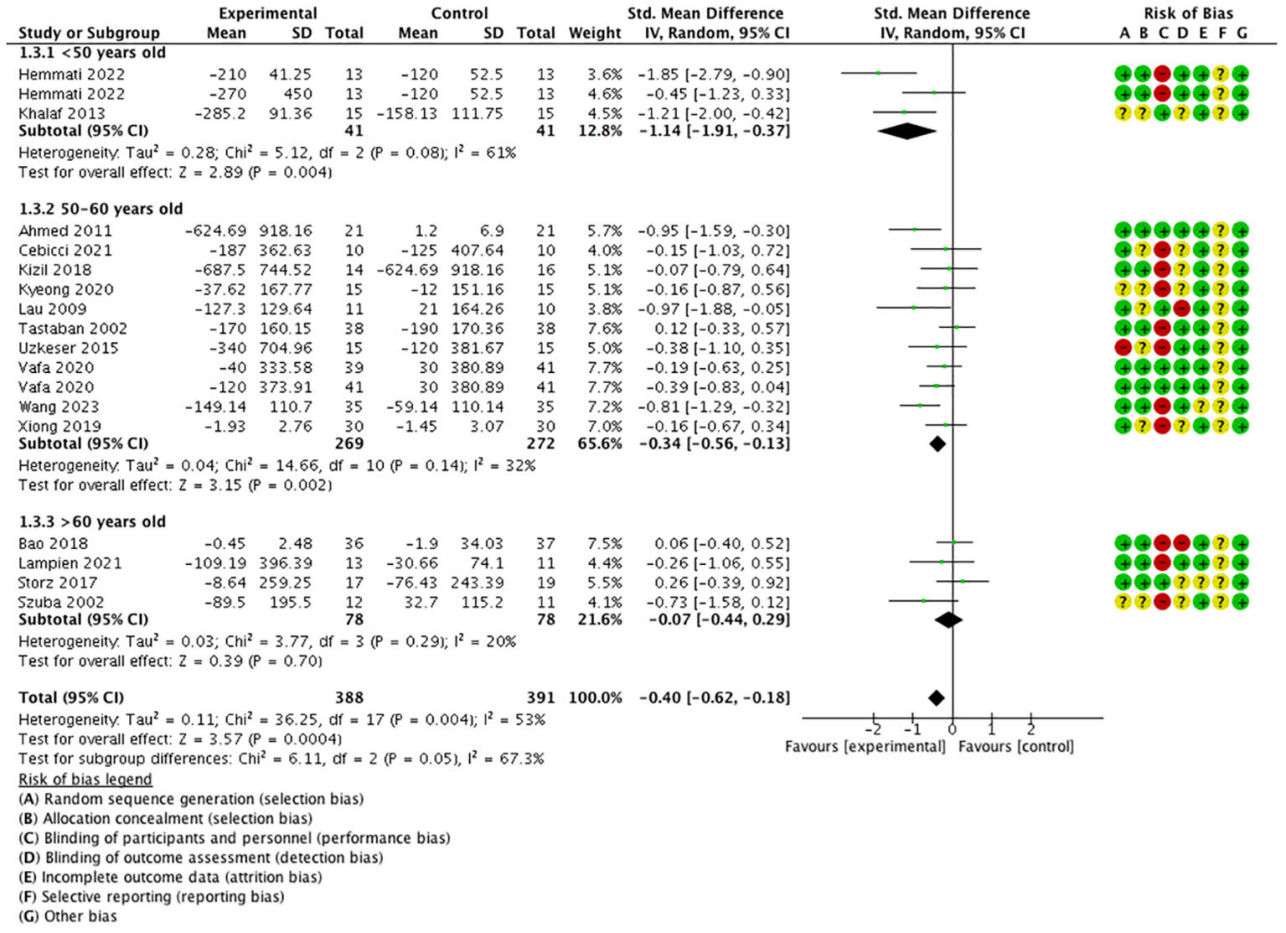
Forest plot of age-grouped analysis.

According to age-grouped data, the age group <50 years old [SMD = −1.14, 95% CI = (−1.91, −0.37), *P* < 0.05, *I* ^2 =^ 61%] and between 50 and 60 years old [SMD = −0.34, 95% CI = (−0.56, −0.13), *P* < 0.05, *I* ^2 =^ 32%] showed a better effect; however, the age group >60 years old [SMD = −0.07, 95% CI = (−0.44, 0.29), *P* > 0.05, *I* ^2 =^ 20%] had a relatively poor effect. It can be found that most heterogeneities come from the age group <50 years old (**Figure 4**).

##### 3.4.2.2 Different treatments

**Figure 5:**
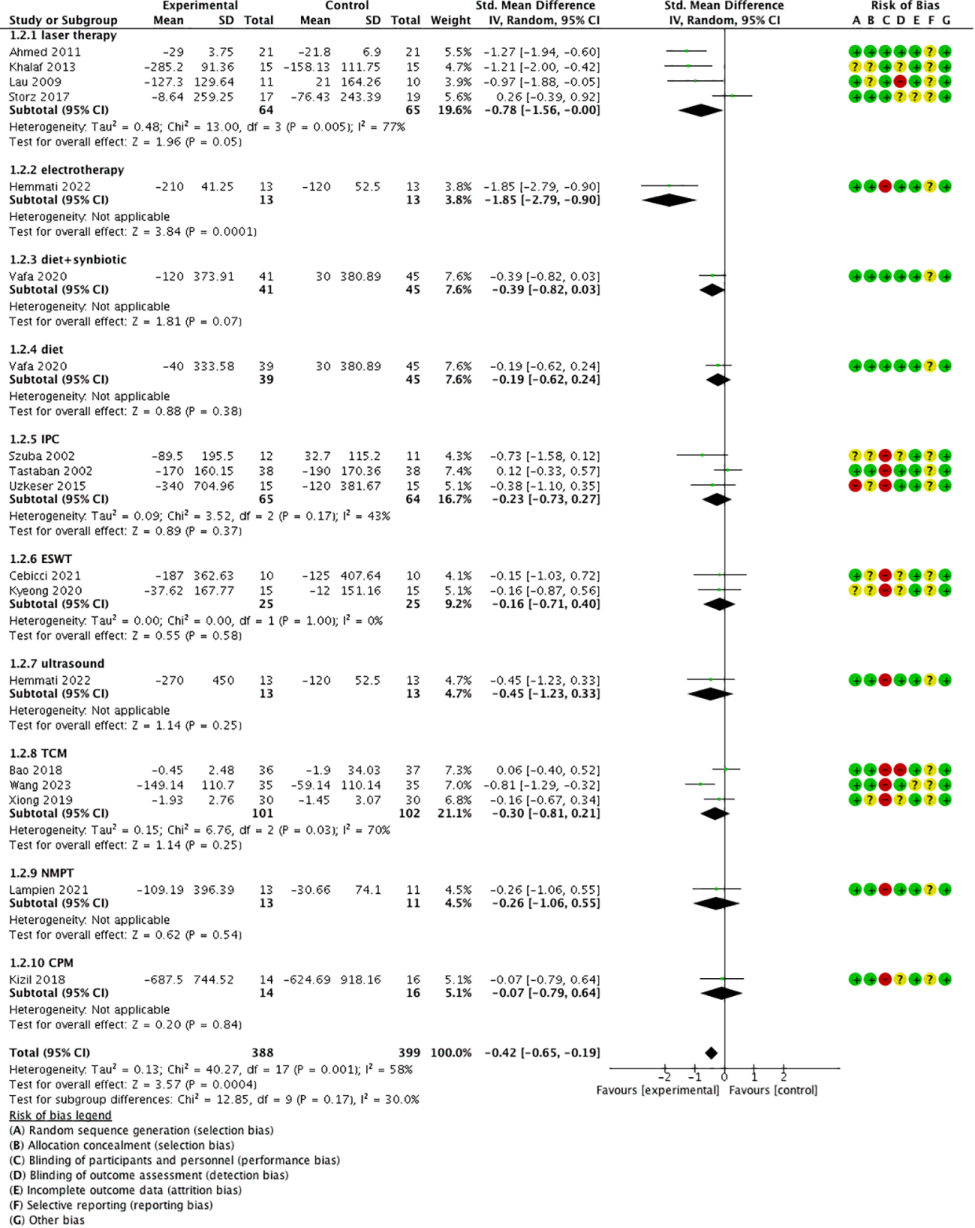
Forest plot of treatment-grouped analysis.

Subgroup analysis based on different treatments showed that laser therapy [SMD = −0.78, 95% CI = (−1.56, −0.00), *P* = 0.05, *I* ^2 =^ 77%] and electrotherapy [SMD = −1.85, *P* < 0.05, 95% CI = (−2.79, −0.90)] had better effect, and diet/diet + synbiotic, IPC, ESWT, ultrasound, TCM (sliding cupping, acupuncture, Tuina combined with moxibustion), NPMT, and CPM did not show a significantly better effect compared with the control group (*P* > 0.05) (**Figure 5**).

##### 3.4.2.3 Dose-grouped and session-grouped laser therapy analysis

**Figure 6:**
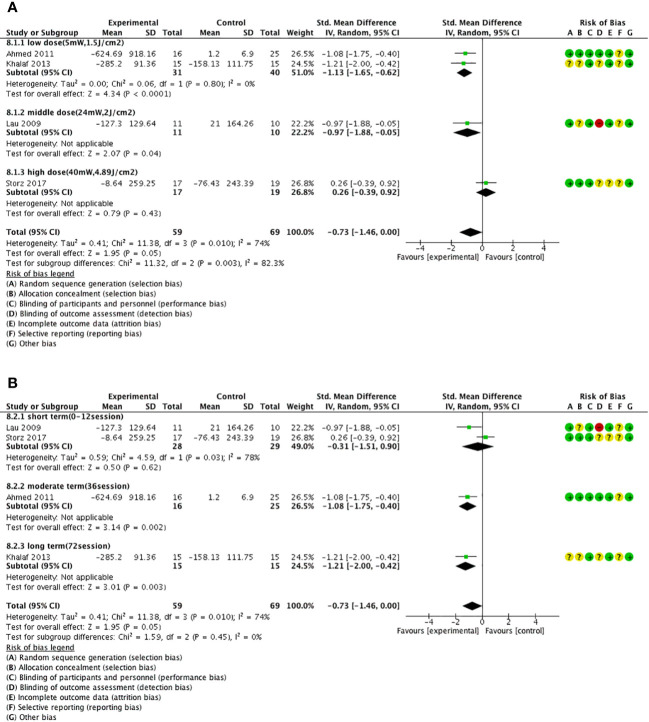
Forest plot of laser therapy subgroup analysis. **(A)** Forest plot of the laser dose–subgroup analysis. **(B)** Forest plot of the laser session–subgroup analysis.

In a dose–subgroup analysis of laser therapy, we found that only low intensity (5 mW, 1.5/cm^2^) [SMD = −1.13, 95% CI = (−1.65, −0.62), *P* < 0.05, *I* ^2 =^ 0%] to moderate intensity (24 mW, 2 J/cm^2^) [SMD = −0.97, 95% CI = (−1.88, −0.05), *P* < 0.05] (**Figure 6A**) and moderate term (36 sessions) [SMD = −1.08, 95% CI = (−1.75, −0.40), *P* < 0.05] to long term (72 sessions) [SMD = −1.21, 95% CI = (−2.00, −0.42), *P* < 0.05] significantly improved lymphedema compared with those of the control group (**Figure 6B**).

### 3.5 Secondary outcome 1: quality of life

**Figure 7:**
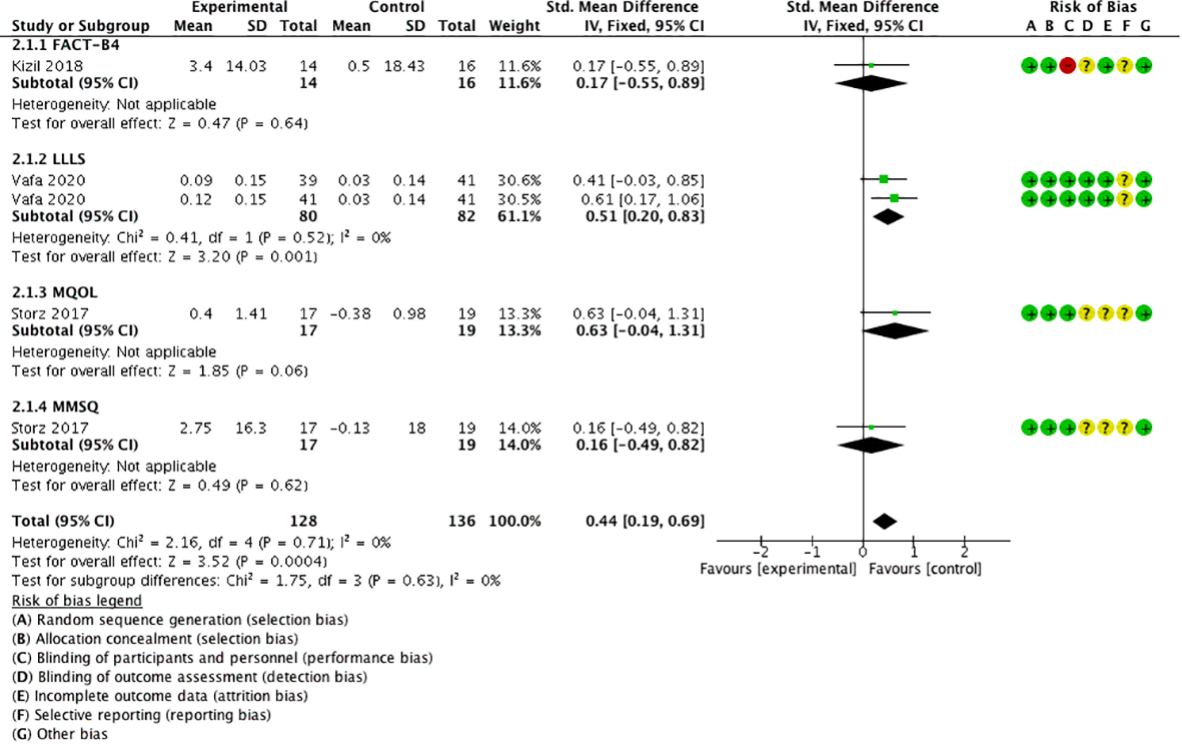
Forest plot of QoL.

Three studies (18, 26, 30) took four comparisons using QoL as the outcome measure, using the scales of Functional Assessment of Cancer Therapy for Breast Cancer (FACT-B4) (30), Lymphedema Life Impact Scale (LLIS) (26), McGill Quality of Life Questionnaire (MQoL), and the German version of the Multidimensional Mood State Questionnaire (MMSQ) (18). The meta-analysis displayed that QoL was significantly higher in the experimental group than in the control group [SMD = 0.44, 95% CI (0.19, 0.69), *I* ^2 =^ 0], and a statistically significant difference was found (*P* < 0.05) (**Figure 7**).

### 3.6 Secondary outcome 2: DASH/QuickDASH

Six studies used the quick disabilities of the arm, shoulder, and hand (QuickDASH) (21, 23, 24) or DASH (17, 30, 31) questionnaire as the outcome (these are self-reported assessment tools that measure physical function and symptoms in individuals with a musculoskeletal disorder of the upper limb). The scores indicated the level of disability and severity, ranging from 0 (no disability) to 100 (most severe disability).

**Figure 8:**
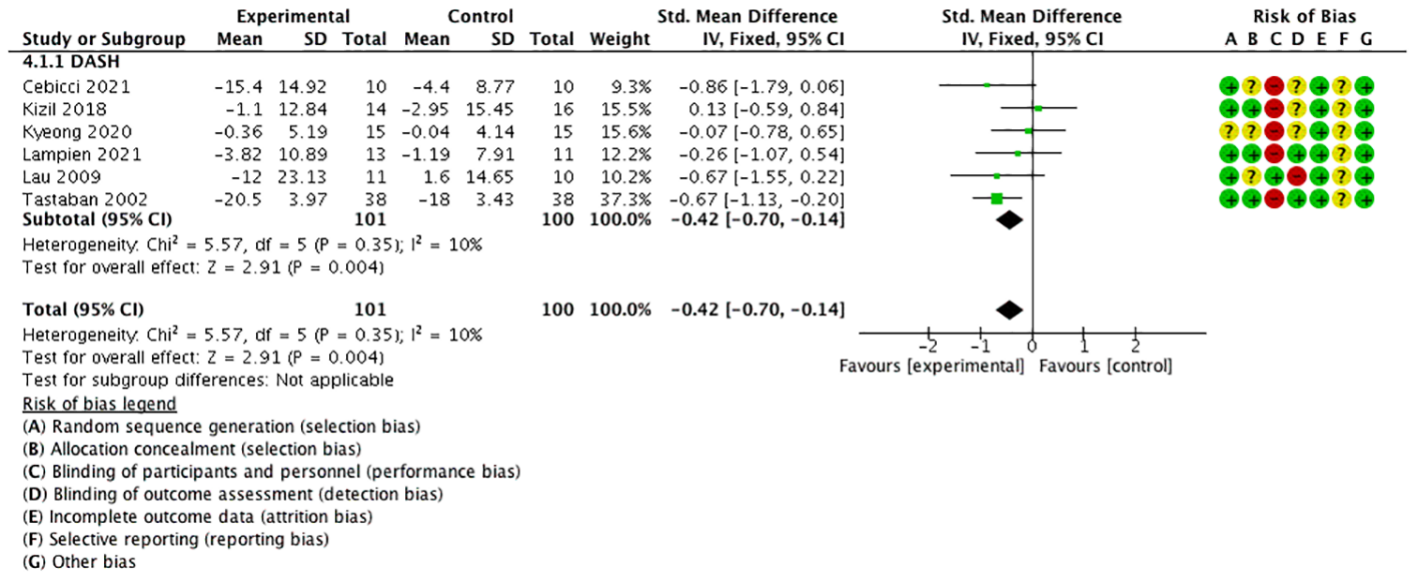
Forest plot of DASH and QuickDASH.

The meta-analysis manifested that QuickDASH/DASH was significantly lower in the experimental group than in the control group [SMD = −0.42, 95% CI (−0.70, −0.14), *I* ^2 =^ 10%], and a statistically significant difference was found (*P* < 0.05) (**Figure 8**).

### 3.7 Secondary outcome 3: VAS for pain

**Figure 9:**
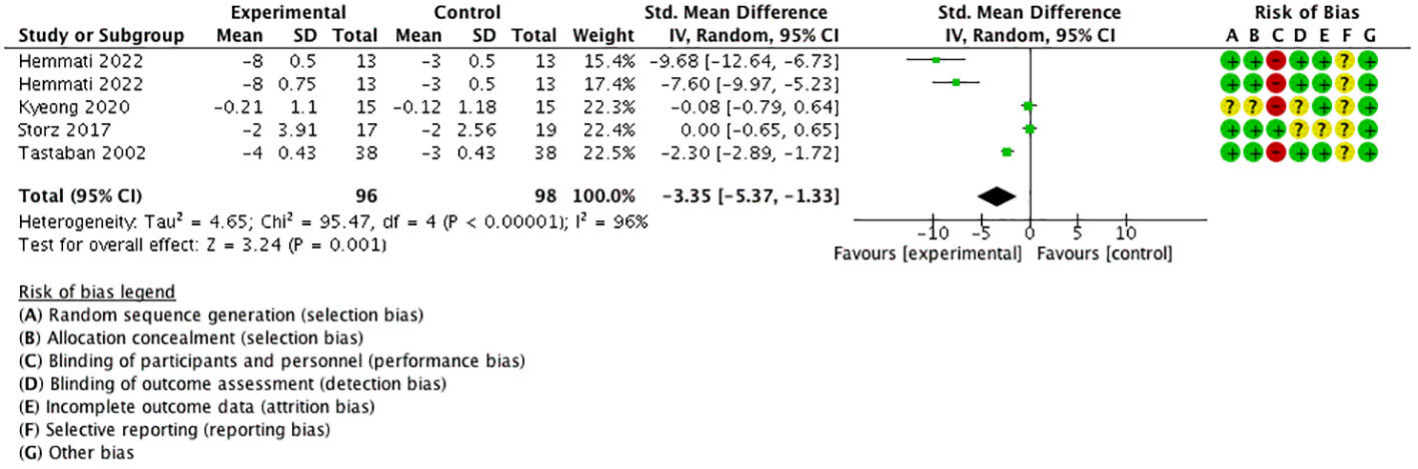
Forest plot of VAS for pain.

Four studies (18, 21, 23, 25) performed five comparisons using the visual analog scale (VAS) for pain as the outcome with a 0–10 numerical rating scale. The meta-analysis proved that VAS for pain was significantly decreased in the experimental group than in the control group [SMD = −3.35, 95% CI (−5.37, −1.33), *I* ^2 =^ 96%], and a statistically significant difference was found (*P* < 0.05) (**Figure 9**).

### 3.8 Adverse events

One study reported the AEs in detail (during acupuncture, bruises 45 cases, hematoma 2 cases, pain 2 cases, skin infection 1 case) and 7 studies reported no AEs (ESWT, IPC, LLLT, NPMT, diet, synbiotic). However, 10 studies did not mention AEs.

## 4 Discussion

### 4.1 Main findings

Our results showed that the addition of certain conservative medical treatments to CDT can exert a better effect in reducing the volume/circumference of the swollen limbs of BCRL patients, as well as relieve pain and disability and also improve the quality of life of the patients. Furthermore, we found among the various treatments that laser therapy and electrotherapy had the best effect in relieving swollen limbs, and we recommend laser therapy of 5–24 mW, 1.5–2 J/cm^2^ intensity, and 36–72 sessions for BCRL patients to achieve the best effect based on the subgroup analysis findings. Moreover, patients <60 years old may show a better effect than elderly patients, indicating the importance for younger patients to receive relative treatments as it will be more efficient and economical.

### 4.2 Strengths and limitations

Our review has several strengths. First, most of the included articles were of moderate to high quality with relatively standardized design, which improved the confidence of the results. Second, the entire search strategy and data analysis process were relatively formal. Third, we included 16 eligible RCTs with 655 participants investigating the additional beneficial effect of conservative medical treatments on swelling, pain, disability, and quality of life. Fourth, this study was conducted using in-depth subgroup meta-analyses to evaluate potential sources of heterogeneity.

However, this study has some limitations. First, some TCM treatments (including acupuncture/sliding cupping) did not show a significant overall effect in our study although some beneficial effects were observed (33), and this may have resulted from the lack of search of Chinese databases such as CNKI, VIP, and SinoMed, which may contain more research about TCM, and the selection and combination of acupoints also play an important role in the effectiveness of treatments. Second, this study was based on study-level data but not on patient-level data, and the analysis was based on small sample sizes, so the results should be interpreted carefully. Third, our study did not include gray literature such as some studies from the National Institutes of Health (NIH) and the World Health Organization (WHO). Fourth, this review just included RCT studies and excluded non-RCTs such as prospective pilot studies, and this might cause an underestimated effect of some treatments. Fifth, the exclusion criteria in the included studies did not mention the history of surgery to treat lymphedema, which may cause some efficacy differences.

### 4.3 Relation to previous studies

Several meta-analyses about BCRL treatment came to the following conclusions: one study concluded that conservative treatment interventions may not meaningfully improve lymphedema volume compared with standard care (34), and this study did not take CDT as the control group, which is the first-line treatment. Another study concluded that acupuncture and moxibustion (33) are effective in treating BCRL; however, different control methods were used; therefore, it is difficult to interpret the conclusions of the study. A third study concludes that ESWT combined with CDT could have significant effects (35); however, some non-RCT studies were included, which might affect the results. The present study, on the other hand, excluded non-RCT studies as the focus was on medical practice; furthermore, various kinds of conservative treatments were included to obtain broader knowledge, and CDT was used as a first-line treatment to improve its practical application value.

### 4.4 Future perspectives

Our findings indicate that it is meaningful to discover and promote conservative medical treatments in clinical practice to better help BCRL patients relieve their symptoms such as swollen limbs, pain, and disability and improve their life quality. However, there is still a lack of consensus on which patients will benefit from each treatment option, and there are no guidelines on the appropriate time to start treatment, which can lead to treatment issues (36).

Low-level laser therapy (LLLT), also called photobiomodulation (PBM), may reduce inflammation, prevent fibrosis, and stimulate lymphangiogenesis (32). Electrical stimulation reduces edema by increasing muscle contraction and can increase lymph and blood flow up to 30-fold; moreover, muscle contraction favors the removal of intercellular proteins (37). However, there are relatively fewer studies about electrotherapy, and future studies can explore more about the efficacy and safety as well as the intervention time of applying electrotherapy.

The selection and combination of conservative medical treatments may play an important role in the effectiveness of treatments. At present, CDT is widely known as the most important treatment for BCRL. Previous meta-analyses (35) concluded that ESWT combined with CDT could significantly improve the volume of lymphedema in BCRL patients but not enough to replace CDT. Our review also supported the idea that a combination of conservative treatments with CDT could provide significant clinical benefits to BCRL patients but cannot replace CDT in treating BCRL.

Moreover, different measurements of the affected limbs by circumference or volume calculated by circumference or water displacement made it difficult to aggregate analyses. In future trials, it may be a better option to report the volume changes by water displacement to uniform the measurement, and water displacement is more direct than calculation by circumference. Conservative rehabilitation interventions need to be continued to develop studies to help guide therapeutic decisions that can promote health-related quality of life in BCRL women (38).

As for safety assessment, no adverse events occur when applying ESWT, IPC, LLLT, NPMT, diet, or symbiotic treatment, and most AEs come from acupuncture, indicating that practitioners should pay more attention in performing acupuncture and poor performance may also reduce the effect of acupuncture.

## 5 Conclusion

In conclusion, our research supports the efficacy of combining conservative medical intervention with CDT over utilizing CDT alone in improving the health status of BCRL patients (reducing lymphedema volume, pain, and functional disability and improving quality of life), particularly with the inclusion of laser therapy and electrotherapy. This combination showed better effects on patients under 60 years old, and laser therapy of low to moderate intensity (5–24 mW, 1.5–2 J/cm^2^) and of moderate- to long-term duration (≥36–72 sessions) showed better effects.

## Data availability statement

The original contributions presented in the study are included in the article/**Supplementary Material**. Further inquiries can be directed to the corresponding authors.

## Author contributions

CD: Conceptualization, Data curation, Formal analysis, Software, Supervision, Writing – original draft, Writing – review & editing. ZW: Writing – original draft, Data curation, Formal analysis, Software. ZC: Writing – review & editing, Software. XZ: Writing – original draft, Funding acquisition, Supervision, Writing – review & editing. CT: Writing – review & editing, Conceptualization, Funding acquisition, Supervision.
