# Supplementary material for: Conservative medical intervention as a complement to CDT for BCRL therapy: a systematic review and meta-analysis of randomized controlled trials
Source: Front Oncol. 2024 Apr 26;14:1361128. doi: 10.3389/fonc.2024.1361128 (PMC11082302; doi:10.3389/fonc.2024.1361128)
Supplement: Supplementary file 1 [file DataSheet_1.docx]

**Appendix 1.** Search strategies

【Pubmed】

"Breast Cancer Lymphedema"[MeSH Terms] OR ("Breast Cancer Lymphedema"[MeSH Terms] OR (("breast neoplasms"[MeSH Terms] OR ("Breast"[All Fields] AND "neoplasms"[All Fields]) OR "breast neoplasms"[All Fields] OR ("Breast"[All Fields] AND "Cancer"[All Fields]) OR "breast cancer"[All Fields]) AND "Lymphedemas"[Title/Abstract]) OR "lymphedema breast cancer"[Title/Abstract] OR "breast cancer treatment related lymphedema"[Title/Abstract] OR "breast cancer treatment related lymphedema"[Title/Abstract] OR "breast cancer related arm lymphedema"[Title/Abstract] OR "breast cancer related arm lymphedema"[Title/Abstract] OR "breast cancer related lymphedema"[Title/Abstract] OR "postmastectomy lymphedema"[Title/Abstract] OR "lymphedema postmastectomy"[Title/Abstract] OR (("Lymphedema"[MeSH Terms] OR "Lymphedema"[All Fields] OR "Lymphedemas"[All Fields] OR "lymphoedema"[All Fields] OR "lymphoedemas"[All Fields]) AND "Postmastectomy"[Title/Abstract]) OR ("Postmastectomy"[All Fields] AND "Lymphedemas"[Title/Abstract]) OR "post mastectomy lymphedema"[Title/Abstract] OR "lymphedema post mastectomy"[Title/Abstract] OR "post mastectomy lymphedema"[Title/Abstract] OR "post mastectomy lymphedemas"[Title/Abstract])) AND ("Therapeutics"[MeSH Terms] OR ("Therapeutic"[Title/Abstract] OR "Therapy"[Title/Abstract] OR "Therapies"[Title/Abstract] OR "Treatment"[Title/Abstract] OR "Treatments"[Title/Abstract])) AND ("Randomized Controlled Trial"[Publication Type] OR "Randomized Controlled Trials as Topic"[MeSH Terms]

【Cochrane】

#1 MeSH descriptor: [Breast Cancer Lymphedema] explode all trees

#2 (Postmastectomy Lymphedema):ti,ab,kw OR (Post-mastectomy lymphedemas):ti,ab,kw OR (Post mastectomy Lymphedema):ti,ab,kw OR (Lymphedemas, Postmastectomy):ti,ab,kw OR (Lymphedema, Postmastectomy):ti,ab,kw (Word variations have been searched)

#3 (Lymphedema, Post-mastectomy):ti,ab,kw OR (Postmastectomy Lymphedemas):ti,ab,kw OR (Post mastectomy Lymphedema):ti,ab,kw OR (Breast Cancer Treatment Related Lymphedema):ti,ab,kw OR (Breast CancerRelated Arm Lymphedema):ti,ab,kw (Word variations have been searched)

#4 (Lymphedema, Breast Cancer):ti,ab,kw OR (Breast Cancer Related Lymphedema):ti,ab,kw OR (Breast Cancer Related Arm Lymphedema):ti,ab,kw OR (Breast Cancer Treatment-Related Lymphedema):ti,ab,kw OR (Breast Cancer Lymphedemas):ti,ab,kw (Word variations have been searched)

#5 #1 OR #2 OR #3 OR #4

#6 MeSH descriptor: [Therapeutics] explode all trees

#7 (Therapy):ti,ab,kw OR (Treatment):ti,ab,kw OR (Treatments):ti,ab,kw OR

(Therapies):ti,ab,kw OR (Therapeutic):ti,ab,kw (Word variations have been

#8 #6 or #7

#9 MeSH descriptor: [Randomized Controlled Trial] explode all trees

#10 (Controlled Clinical Trials, Randomized):ti,ab,kw OR (Trials, Randomized Clinical):ti,ab,kw OR (Clinical Trials, Randomized):ti,ab,kw (Word variations have been searched)

#11 #9 OR #10

#12 #5 AND #8 AND #11

【Embase】

#1 "breast cancer lymphedema":ab,ti OR “breast cancer lymphoedema”:ab,ti OR “breast cancer related lymphoedema”:ab.ti OR “post-breast cancer lymphedema”:ab.ti OR “post-breast cancer lymphoedema”:ab.ti

#2 “breast cancer-related lymphedema”/exp

#3 #1 OR #2

#4 “therapy”/exp

#5 ‘treatment effectiveness’:ab,ti OR ‘disease therapy’:ab,ti OR ‘disease treatment’ ab,ti OR ‘diseases treatment’:ab,ti OR ‘disorder treatment’: ab,ti OR "disorders treatment"ab,ti OR ‘efficacy therapeutic :ab,ti OR ‘iliness tretment’:ab,ti OR ‘medical therapy’:ab,ti OR 'medical treatment':ab.ti OR ‘multiple therapy’:ab,ti OR ‘poly therapy’:ab,ti OR 'somatotherapy':ab.ti OR "therapeutic action":ab,ti OR ‘therapeutic efficacy’:ab,ti OR ‘therapeutic trial’:ab,ti OR ‘therapeutic trials’:ab,ti OR"therapeutics; therapy medical":ab,ti OR “treatment efficacy”:ab,ti OR 'treatment, medical":ab.ti OR “combination therapy”:ab,ti

#6 #4 OR #5

#7 ‘randomized controlled trial’/exp

#8 ‘controlled trial,randomized’:ab,ti OR ‘randomised controlled study’:ab,ti OR ‘randomised controlled trial': ab.ti OR “randomized controlled study”:ab.ti OR “trial, randomized controlled”:ab.ti

#9 #7 OR #8

#10 #3 AND #6 AND #9

【Web of science】

1: (TS=(BCRL)) OR TS=(breast cancer related lymphedema)

2: ((TS=(therapy)) OR TS=(treatment)) OR TS=(Therapeutic)

3: (TS=(Randomized Controlled Trial)) OR TS=(RCT)

4: #3 AND #2 AND #1
